# BAMSE: Bayesian model selection for tumor phylogeny inference among multiple samples

**DOI:** 10.1186/s12859-019-2824-3

**Published:** 2019-06-06

**Authors:** Hosein Toosi, Ali Moeini, Iman Hajirasouliha

**Affiliations:** 1 Institute of Biochemistry and Biophysics, University of Tehran, Tehran, Iran; 2 School of Engineering Sciences, College of Engineering, University of Tehran, Tehran, Iran; 3 Institute for Computational Biomedicine, Weill Cornell Medicine, New York, NY USA; 4 Department of Physiology and Biophysics, Weill Cornell Medicine, New York, NY USA; 5 Englander Institute for Precision Medicine, Weill Cornell Medicine, New York, NY USA; 6 The Meyer Cancer Center, Weill Cornell Medicine, New York, NY USA

**Keywords:** Bayesian model selection, DNA sequencing, Tumor phylogeny, Computational cancer genomics, Next generation sequencing, Tumor heterogeneity, Clonal evolution

## Abstract

**Background:**

Intra-tumor heterogeneity is known to contribute to cancer complexity and drug resistance. Understanding the number of distinct subclones and the evolutionary relationships between them is scientifically and clinically very important and still a challenging problem.

**Results:**

In this paper, we present BAMSE (BAyesian Model Selection for tumor Evolution), a new probabilistic method for inferring subclonal history and lineage tree reconstruction of heterogeneous tumor samples. BAMSE uses somatic mutation read counts as input and can leverage multiple tumor samples accurately and efficiently. In the first step, possible clusterings of mutations into subclones are scored and a user defined number are selected for further analysis. In the next step, for each of these candidates, a list of trees describing the evolutionary relationships between the subclones is generated. These trees are sorted by their posterior probability. The posterior probability is calculated using a Bayesian model that integrates prior belief about the number of subclones, the composition of the tumor and the process of subclonal evolution. BAMSE also takes the sequencing error into account. We benchmarked BAMSE against state of the art software using simulated datasets.

**Conclusions:**

In this work we developed a flexible and fast software to reconstruct the history of a tumor’s subclonal evolution using somatic mutation read counts across multiple samples. BAMSE software is implemented in Python and is available open source under GNU GLPv3 at https://github.com/HoseinT/BAMSE.

## Background

In his seminal paper, Peter Nowell [1] proposed the clonal evolution theorem. He hypothesized that single tumors consist of subclones with distinct genetic makeup, all descending from an initiating cancerous *founder cell*. These subclones are subject to Darwinian evolution in their environment. i.e. they may expand with rapid cell divisions, new subclones appear as mutations accumulate in earlier ones and subclones may vanish as a result of competition with each other. With the advent of cost-effective and massively parallel DNA sequencing technologies, tumor genomes and often their matched normal genomes are now being sequenced routinely. Quantifying distinct subclones in a tumor and their genetic composition has been shown to be of clinical value in several recent studies. (e.g. [2, 3]). Having robust, fast and scalable automated methods for inferring clonality in tumor samples is, thus, extremely valuable.

Although single-cell sequencing (SCS) is gaining popularity in recent years, the vast majority of cancer sequencing experiments are still performed on *bulk* samples which are indeed mixtures of a large number of cells. This is because (a) SCS experiments are expensive and still not feasible for large scale high-throughput screenings, and (b) SCS library preparation is complex and often introduces a high-level of noise and missing signals.

Bulk tumor DNA sequencing and subsequent downstream analyses (e.g. variant calling) help us measure somatic variations in tumor samples. These somatic variations can be in the form of single nucleotide variants (SNV), insertions or deletions (Indels), copy number variations (CNVs) or complex structural variations (SVs). Similar to several recent studies that aim to quantify tumor evolution [4–9], we also focus on SNVs, primarily because it is the class of somatic variants that can be measured relatively in a robust way, using the current technology. Using standard short-read sequencing, the number of reads supporting the reference allele and the number of those supporting the variant allele at each somatic variant locus, gives us an estimate on the variant allele fraction (VAF) of each somatic SNV. Given these measurements (i.e. the variant allele fraction of each somatic SNV in each of the given samples), the intra-tumor heterogeneity deconvolution problem is to infer the normal contamination, number of subclones and mutations falling in each of them, and reconstructing the tumor phylogeny relating these subclones.

Practically, a large number of possible combinations of *valid* tree topologies, along with the sequencing noise make this problem impossible to solve exactly and several variations of this problem have been computationally proven to be indeed NP-hard [5, 10]. Several assumptions are often made to limit the solution space with biologically relevant constraints. The most popular constraint is the Infinite Site Assumption (ISA), where it is assumed that when a nucleotide (cite) is mutated, it will be extremely unlikely to mutate again. Thus, somatic mutations that are shared among different subclones must share a common ancestor in the underlying tumor phylogeney tree. This also means that an SNV in a subclone is present only in that subclone and the subclones descending from it. Other common assumptions are sparsity and shallowness [11]. Sparsity means favoring solutions that have unobserved subclones and shallowness is favoring the trees with lower depth. In this paper, we assume the ISA and use parameters in our model to control sparsity.

Existing computational tools proposed to solve the intra-tumor heterogeneity problem may output the most likely solution or a set of solutions. An overview of existing tools is available in [12, 13]. The procedure for these software packages usually includes these steps:

1. The percentage of cells having each mutation is estimated: in the simplest form, assuming diploid genomes, twice the variant allele fraction (VAF) is used. If reliable copy number data from sequencing or microarray experiments are available, these data are used along with the read counts to estimate the fraction of cells having each of the mutation.
2. The mutations are clustered based on similarities of their cellular prevalence, each cluster representing a subclone. Various clustering methods have been used in literature [4, 6, 9, 14].
3. Normal contamination and evolutionary relationships are inferred using the estimated percentage of cells in each subclone: The highest prevalence in the subclones is considered to be the tumor content. Tree reconstruction algorithms are used to output possible trees topologies.

In this paper we propose a novel Bayesian model selection based tool, BAMSE, as a new powerful alternative to reconstruct tumor phylogenies and find the more probable tumor phylogeny when multiple heterogeneous samples are given.

We compared the performance of BAMSE with three state-of-the-arts software, LICHeE [4], Ancestree [10] and PASTRI [15] that are capable of handling multiple samples. LICHeE first groups subsets of somatic SNVs that have similar patterns of presence/absence as well as similar variant allele fractions (VAF) across samples. Then, it constructs a network, named as *constrained network*, which embeds all ancestral relationships among clusters of somatic SNVs. Finally, using a variation of spanning tree search algorithms, LICHeE identifies tumor phylogeny trees. Ancestree [10] formalizes the tumor phylogeny reconstruction problem as a matrix factorization problem (i.e. variant allele frequency factorization) and gives an ILP solution for it. Ancestree also uses a spanning tree search algorithm to make the method scalable and capable of handling noise. A very recent tool to address this problem, PASTRI [15] uses integration by importance sampling and also does not require an MCMC sampling. PASTRI marginalizes over cluster assignments but then has to use costlier integrations via importance sampling than our approach. PASTRI also depends on other clustering tools to obtain the number of subclones and the proposal distribution for importance sampling. While BAMSE goes for a built-in costlier clustering approach, it has an easier integration and is completely independent from other software.

Two other notable methods that deal with multiple samples are PhyloSub [6] and CITUP [7]. PhyloSub [6] uses a Bayesian non-parametric model and a Markov chain Monte Carlo (MCMC) sampling to solve this problem. The MCMC sampling part in PhyloSub makes the method slower than a combinatorial method such as LICHeE [4]. CITUP [7] uses an elegant exact Quadratic Integer Programming formulation to solve this problem, but does not leverage the read counts information. Similar to PhyloSub, our method BAMSE is probabilistic but does not require a computationally expensive MCMC sample. BAMSE can perform the MCMC sampling with much fewer parameters and converge faster, or alternatively BAMSE can just use a K-means based approach with no need of a computationally expensive MCMC sampling.

Our paper is organized as follows: the methods section describes our proposed model and method in detail. In the “” section, we test our method on synthetic and real tumor data sets and highlight why our method is superior to the existing tools. Finally, in the last section, we discuss the results and the advantage of our approach over existing tools.

## Implementation

### The Bayesian Model

We first explain the details of our model for the single sample scenario. We then show how to extend it for multiple samples.

Given *N* observed mutations in one tumor sample, we aim to score any given model for subclonal evolution of the tumor. A model $\mathcal M$ first clusters the observed mutations into $K_{\mathcal M}$ subclones labeled $1,2,\dots, K_{\mathcal M}$ and then places each subclone on a corresponding node of a rooted labeled tree $T_{\mathcal M}$. This tree describes the evolutionary relationships among subclones; if a node *i* is a child of another node *j*, it implies that the subclone *i* has evolved directly from the subclone *j*.

We use an integer vector $\mathbf {c}_{\mathcal M}$ to denote the clustering for model $\mathcal M$: $c_{\mathcal M_{n}} = k$ simply means that the *n*th mutation is assigned to the *k*th subclone.

For model $\mathcal M$ with *K* subclones, there are *K* parameters that define the fraction of cells for each subclone. Let *u*_*k*_ be the fraction of cells in subclone *k* and let *f*_*k*_ be the fraction of cells carrying mutations of subclone *k*. Because of ISA constraints, we have:

1 $$  u_{k} = f_{k}-\Sigma_{i \in \text{children of node k}} f_{i}  $$

if we make a *K*×*K* binary matrix *B* where *B*_*i,j*_=1 iff *j* is a descendant of *i* or is equal to *i*. we have:

2 $$  \mathbf{f} = B \times \mathbf{u} \\ \mathbf{u} = B^{-1} \times \mathbf{f}  $$

Note that the above observation is equivalent to the formula derived in [10] to describe the relation between the subclone frequency *F* and subclone usage *U*. To place a prior over model parameters, we suppose that the subclone fractions *u*_*k*_ are drawn from a prior over inside the unit simplex (it is obvious that the sum $\sum \nolimits _{k=1}^{K} u_{k}$ cannot exceed 1). The distribution may not necessarily be uniform as BAMSE can handle any prior whose distribution function is a multiplicatively separable function of *u*_*k*_s. This is a small constraint as Dirichlet distribution, the only popular distribution over inside the unit simplex, is among the allowed priors. So the prior for subclone cellular fractions is:

3 $$  Pr(\mathbf{u}={u_{1},u_{2},\dots,u_{K}}) = \prod_{k=1}^{K} p_{k}(u)  $$

As mentioned above, a model is a clustering of mutations followed by an arrangement of these clusters in a rooted tree. For Bayesian analysis, we need to define a prior distribution for such models. Here we use an extension of the Hierarchical Uniform Prior (HUP) of [16] for the purpose of clustering mutations into subclones. HUP places equal priors for each configuration of clustering *N* objects into *K* clusters. For example, with 5 objects and 3 clusters, there are two configurations: {3,1,1} and {2,2,1}. Of 25 ways to cluster five objects in three clusters, 10 are from the first configuration and 15 are from the second one. With HUP the priors are assigned such that the sum of prior probability for all clusterings of each configuration is indeed the same.

We use a similar approach for priors over trees. We design a prior over the set of labeled trees that assigns equal prior to unlabeled trees with the same number of nodes. For example with *K*=3, there are 9 labeled trees: 6 of them are linear and 3 are branching. As unlabeled configurations are to get equal priors, the prior probability for the 6 linear trees will be $\frac {1}{12}$ and for the remaining tree it will be $\frac {1}{6}$. If we had defined a uniform prior over the labeled trees, then the linear configuration would get twice as much probability as the branching topology.

Tree Structured Stick Breaking (TSSB) [17] is an extension of Dirichlet process prior into two dimensions (i.e. depth and breadth of the tree) and has been successfully used by Phylosub[6], PhyloWGS[14] and BitPhylogeny [18] for study of intra-tumor heterogeneity. While BAMSE can also work with TSSB, we avoid it here because we would not have equal prior over tree topologies.

For Bayesian model selection, we also need to compute the probability of observed data under each model. BAMSE just requires an explicit formula for the probability of observed data for each mutation *n*, given *f*_*n*_, its cellular frequency. There are several model based formulas to compute this probability, each using the available information about read counts, sequencing error and copy number variations at the variant locus. For example, in a diploid genome without copy number variations, we can use the following formula for the probability of observing *d* total reads and *v* variant reads for a mutation present in fraction *f* of the cells:

4 $$  \begin{aligned} Pr(v|f) \propto \left(\frac{f}{2}\frac{e}{3}+ \left(1-\frac{e}{3}\right)\left(1-\frac{f}{2}\right)\right)^{v} \\ \left(\left(1-\frac{f}{2}\right)\frac{e}{3}+ \left(1-\frac{e}{3}\right)\frac{f}{2}\right)^{d-v} \end{aligned}  $$

Note that, there are more complex formulas that can also take copy number variations and over-dispersion into account, while there are simpler methods that do not explicitly use the sequencing error. As long as there is an explicit formula for *Pr*(**D**_*n*_|*f*_*n*_) relating data for each mutation *n* to its cellular frequency, BAMSE is easily applicable. With clustering mutations into subclones, we assume that the mutations in each subclone have the same frequency. Thus, given *f*_*k*_, the probability of data for the mutations falling in subclone *k* is:

5 $$ s_{k}(f_{k}) = Pr(\mathbf{D}_{\mathbf{c}_{n}=k}|f_{k}) = \prod_{n | \mathbf{c}_{n}=k} Pr(\mathbf{D}_{n}|f_{k})  $$

We can compute the model posterior as follows:

6 $$ {\begin{aligned} &Pr(\mathcal{M}|\mathbf{D}) \propto Pr(\mathcal{M}) Pr(\mathbf{D}|\mathcal{M}) \\ &\quad= Pr(Tree)\idotsint\displaylimits_{S_{\mathcal{M}}} \prod_{k=1}^{K} s_{k}(f_{k})p_{k}(f_{k}-\Sigma_{i \in \text{children of node k}} f_{i}) \, \mathrm{d}f_{1} \mathrm{d}f_{2} \dots \mathrm{d}f_{K} \end{aligned}}  $$

**Fig. 1:**
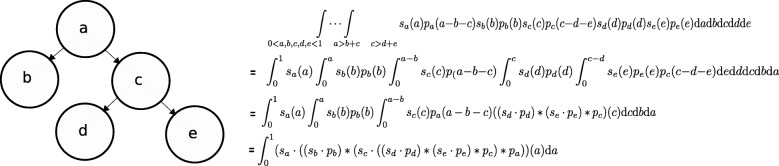
An example explaining the integration method over valid values for subclone fractions given the tree structure. The calculations on the right show how a series of multiplications and convolutions are used to compute the integral in Eq. 6 for the tree on the left

$S_{\mathcal {M}}$ is the region where *f*_1_,*f*_2_,…,*f*_*k*_ are consistent with ISA constraints for model $\mathcal {M}$, that is $f_{k} \geq \sum \nolimits _{i \in children(k)} f_{i}$. Equation 1 was used to write the prior over *u*_*k*_s as a function of *f*_*k*_s. We noticed that the integrals in Eq. 6 can be calculated with a series of convolution and multiplication operations over the functions *s*_*k*_ ans *p*_*k*_. This is because the integrand is a multiplication of univarate factors, and we can start with factors associated with leaf nodes and compute the integral working up the tree. An example is shown in Fig. 1. If function $s_{k}^{*}$ is defined over [0,1] for each node *k* of the tree as:

7 $$ {\begin{aligned} s_{k}^{*}(f)= \left\{\begin{array}{ll} s_{k}(f) \cdot p_{k}(f) & \text{k is a leaf node} \\ s_{k}(f) \cdot(\text{cumconv}({s_{i}^{*} | i \in \mathrm{children \, of \, k}}) * p_{k}(f)) & \text{k is an internal node} \end{array}\right. \end{aligned}}  $$

where cumconv stands for cumulative convolution of elements in a set of functions, it is easily shown that $\int _{0}^{1} I_{r}(f) df$ where *r* is the index of root node equals the integral in Eq. 6, as also shown in the example in Fig. 1.

BAMSE can take copy number variations (CNVs) into account by incorporating copy number information in the frequency probability distribution for mutations that fall within a CNV region. However, when CNVs are not present, we can make assumptions and approximations to make a faster inference. For example, if the logarithm of both *s*_*k*_ and *p*_*k*_ functions are concave, the logarithm of the likelihood function (the integrand in Eq. 6) is also concave and we use convex optimization to find the values for subclone fractions that maximize the likelihood function over the convex simplex defined by each tree. With formulas used here for *s*_*k*_s and *p*_*k*_s, it is easy to show that the logarithm of *s*_*k*_s are always concave and the logarithm of the prior is concave when all the Dirichlet parameters are larger than one. CVXPY [19] is used for convex optimization in our implementation.

### Multiple samples

Our analysis is easily extended to include multiple samples. For each model, the integral in Eq. 6 is computed for each sample separately, their cumulative product multiplied by the model prior is proportional to the multi-samples posterior for that model.

### Absence-presence of subclones in samples

With real tumor data, especially data including distant metastasis, each sample includes a subset of the subclones, and theres is a good chance that several subclones are absent in each sample. To account for this, we can use priors that allow sparsity for subclone fractions. A Dirichlet distribution with parameters smaller than one, or a modification of the Dirichlet distribution [20] can be used for this purpose.

### Searching for good models

When *N* and *K* are small, we can indeed test all the models (i.e. combinations of partitioning N into K clusters and rooted labeled trees with K nodes). However, this is often impractical for real data sets. When there is no copy number variants (CNVs) along with Eq. 4, we propose a fast algorithm to find high probability models. We note that when there is no CNV, *s*_*k*_(*f*) are unimodal and we can use K-means to find candidate high probability clusters. For each clustering of the mutations with *K* clusters, there are *K*^*K*−1^ models, and we can approximate average model posterior probability with $I\frac {\text {\# unlabeled trees with K nodes}}{K^{K-1}}$ where *I* is the integral of $\prod s_{k}$ over the unit hypercube. BAMSE uses K-means clustering for a range of *K* (user defined, between 1 and 15 by default) and computes the approximate average probability for each one, then selects the three *K* with best average scores for the next step. In the next step, for each candidate *k*, K-means is ran multiple times and for each clustering, the posterior probability is computed. if *K*<6 we calculate the posterior for all trees, but for *K*≥6 we use Algorithm 1 to filter out high probability trees. The top models from this step are returned as output.

BAMSE uses the Algorithm 1 to find the trees with minimal violation of ISA constraints. Algorithm 1 takes the mean VAF for the clusters across samples, *E*_*k,m*_ and a threshold *δ* and finds all trees that the sum of ISA violations defined as $\sum \nolimits _{m=1}^{M} \sum \nolimits _{k=1}^{K} min(0,E_{k,m}-\sum \nolimits _{\mathrm {i} \in \textrm {children of k}} E_{i,m})$ is not less than *δ*. The algorithm starts with each cluster as the root node and generates all possible trees node by node. If no tree is found, the threshold is reduced.


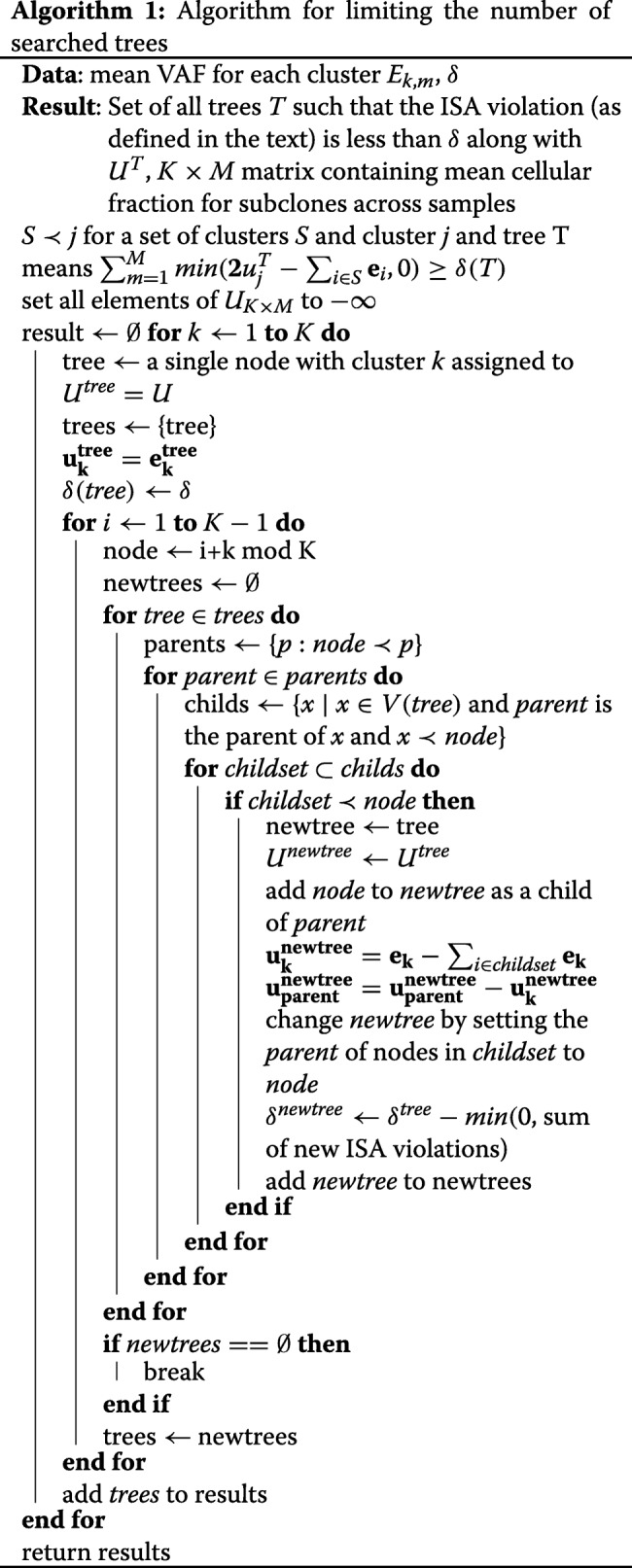


Note that K-means with a range of values for K is used to propose starting points for Algorithm 1, so there are usually trees with different K present in the output.

## Results

In this section, we used simulated and real data sets to highlight the performance of BAMSE.

### Simulated data

BAMSE was benchmarked with Ancestree [10], LICHeE [4] and PASTRI [15]. The following three sets of simulations were considered:

1. **Varying number of subclones**: 80 mutations in 3 samples with sequencing depth 500, varying the number of subclones between 6 and 10
2. **Varying number of samples**: 80 mutations in 8 subclones with sequencing depth 500, varying the number of samples between 2 and 6
3. **Varying sequencing depth**: 80 mutations in 8 subclones across 5 samples with sequencing depth in {300,500,700,900,1100}

Fifty simulations were run for each parameter setting. Four measures were used for the purpose of comparison:

1. **Subclone Fraction Squared Error**: Sum elementwise squared error between the matrices *U* for ground truth and the solution.
2. **Correctly Inferred Relationships**: The percentage correctly inferred evolutionary relationships between all pairs of mutations (co-clustered, parent-child, ancestor-descendant).
3. **Do Tree Structure Match?**: Binary indicator for whether the tree in the solution is the same tree that was used to generate simulated data.
4. **Runtime** Algorithm time to finish in seconds.

**Fig. 2:**
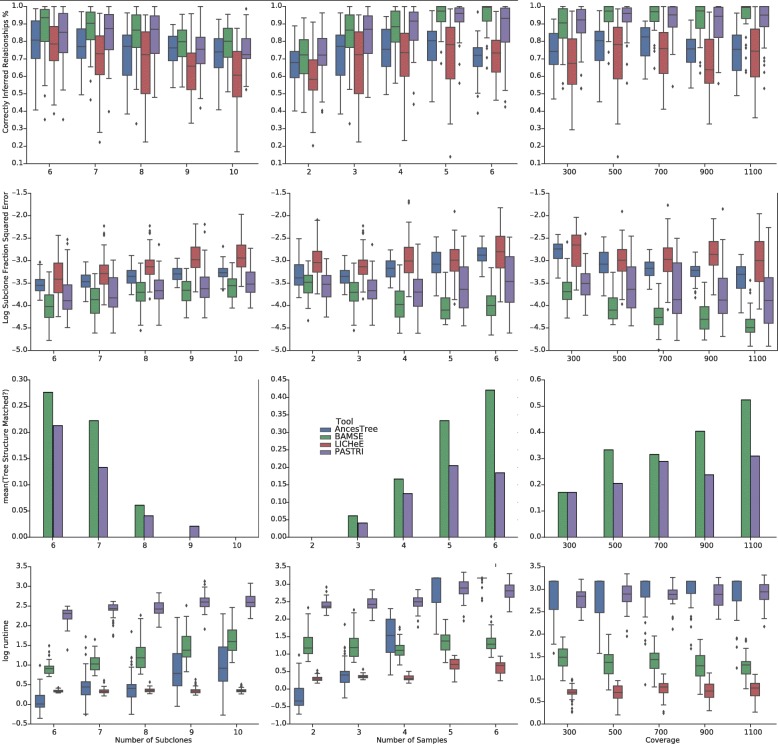
Benchmarking with simulated data results. Comparison of BMASE, Ancestree, LICHeE and PASTRI on simulated datasets. Refer to the “” section for information on the comparison metrics

To generate simulated data, tree typology and clustering configuration were selected uniformly at random, cell fractions drawn from a uniform Dirichlet distribution and then read counts for each mutation were generated using Eq. 4. BAMSE was run with default parameters and without the absent-present pattern. For LICHeE runs, parameters MaxVAFabsent and MinVAFpresent were set depending the coverage and error rate as outlined in the documentation. Ancestree was run with default settings and time limit of 1500s. Ancestree finished running or had a solution before that limit in all simulations. Sciclone [21] was used to generate proposal distributions for PASTRI. As Ancestree and LICHeE may drop some mutations in the solution, the mutations present in the output of all methods were used for benchmarking. The results are shown in Fig. 2.

LICHeE first clusters mutations based on their absent-present pattern in the samples, so it best performs when there are more samples. Ancestree runs fast and accurately with a few samples, however the running time increases drastically when the number of samples increases, which makes it less scalable than BAMSE. For *M*=2 Ancestree runs in less than one second, while for just *M*=6, the algorithm is often terminated with the imposed time limit. As highlighted in the figures, BAMSE consistently performs best among other methods.

For the third measure (Do Tree Structure Match?) the results for BAMSE and PASTRI are shown. The results for the other tools were excluded because they are combinatorial and do not assume a uniform prior over unlabeled tree, and thus had much lower exact mach rate. In particular, LICHeE reports every single lineage tree that satisfy its evolutionary constraint and it would be hard to compare its results according to this measure.

The results show that BAMSE’s performance is not affected by the number of samples and is consistently very good. Another clear advantage of BAMSE is that it always gives better estimates of subclone fractions. This is because BAMSE solves an exact optimization problem in order to find the fractions, while Ancestree solves a linear approximation of the problem.

### ccRCC Data

**Fig. 3:**
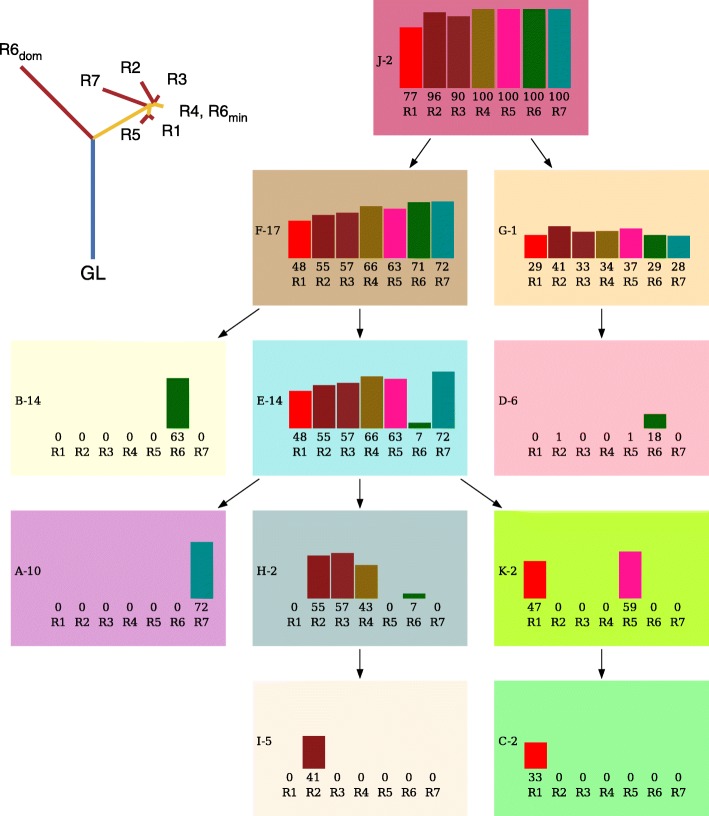
Benchmarking with ccRCC data left: Phylogeny tree for the EV005 patient made by the authors of [22] using extensive manual work in conjunction with traditional phylogeny methods. right: BAMSE output for the same patient. Each node of the tree represents a subclone, named by a letter followed by the number of mutations that fall in it. The bars show the percentage of cell carrying the mutations of that subclone

We also ran BAMSE on a real data set obtained from a study on clear cell renal cell carcinoma [22]. In this study, multi-region deep exome sequencing is used to detect somatic mutations and build the tumor phylogeny tree leveraging parsimony principles and extensive manual work. BAMSE could easily detect high probability models on this data. The results for patient EV005 of the study are shown in Fig. 3, while results of other patients are presented in the Supplemental Materials (see our GitHub repository). As can be seen in Fig. 3, the output of BAMSE and the tree reported in [22] are almost identical. The samples that are neighbors in the phylogenetic tree on left have more subclones in common. There are, however, a few notable differences. The tree reconstruction approach used in [22] considers samples as a whole or breaks them down into dominant and minor fractions and builds a tree, while BAMSE models the samples as sparse collections of subclones in the tumor that comply with ISA constraints. A visible difference in this case (EV005) is that BAMSE breaks the *R*6_*d*_*om* subclone into two additional subclones with 18 and 63 percent cell fractions. When we checked the input read counts, the VAF for mutations observed only in *R*6 are 23,30,14,26,34,39,36,29,27,15,59,15,28,29,40,9,34,31 percent respectively. Using beta mixture regression analysis, These numbers further support the solution obtained by BAMSE, which clustered them into two distinct subclones (*p*-value:0.046). Besides BAMSE has detected a subclone (F) with zero subclone utilization *u*.

## Discussion

In this paper we introduced BAMSE, a novel method that uses Bayesian model selection to infer the evolutionary history of somatic mutations using single or multiple tumor samples form the same cancer patient.

BAMSE has advantages over current existing tools for deconvolution of tumor subclones. It directly uses read counts for the computations, where tools like LICHeE [4] or CITUP [7] just use the pre-computed VAFs. BAMSE is indeed more scalable than methods such as [10, 14], whose running time substantially increases when the number of samples grows. PASTRI [15] is similar to BAMSE in the sense that it also uses integration over simplices and tests unlabeled trees. In contrast with our approach, PASTRI marginalizes over cluster assignments and then has to use costlier integrations via importance sampling. PASTRI also depends on other clustering tools and software, while BAMSE handles all procedures internally.

While in this paper we did not involve copy number variations for the sake of a fast approximate algorithm, in the presence of copy number variations, an MCMC based inference can be indeed used along with BAMSE. We plan to explore this in a feature work. For handling copy number variants, there are two key differences between potential additions to BAMSE and PhyloWGS: a) the *prior* used in these two methods and b) the *variables* they use for inference. BAMSE uses an extension of Hierarchical Uniform Prior assigning a uniform prior for all clustering and tree configurations with the same number of components, which is not the case with TSSB. Since usually we are looking for the topology of the infered trees, BAMSE’s prior is more suitable. Each Markov chain state in PhyloWGS includes variables for subclone frequency and these are updated using Metropolis-Hastings iterations with a Dirichlet distribution proposal. With BAMSE, these variables are integrated out and, thus, will lead to much faster convergence.

## Conclusions

BAMSE considers all popular assumptions for solving the intra-tumor heterogeneity problem, and can also involve copy number variations in principal. We demonstrated that BAMSE is robust and performs very accurately when tested on both simulated and real data. We highlighted that BAMSE performs better than several state-of-the-arts tools. In addition to CNVs, natural future work is to consider other forms of somatic mutations such as complex structural variants in our model.

## Abbreviations

ISA: Infinite sites assumption
